# Impact of L-Citrulline Supplementation and HIIT on Lipid Profile, Arterial Stiffness, and Fat Mass in Obese Adolescents with Metabolic-Dysfunction-Associated Fatty Liver Disease: A Randomized Clinical Trial

**DOI:** 10.3390/nu17030402

**Published:** 2025-01-23

**Authors:** Alan Arturo Rodríguez-Carrillo, Mario Ramón Espinoza-Vargas, Katya Vargas-Ortiz, Lorena del Rocío Ibarra-Reynoso, Monserrat Olvera-Juárez, Armando Gómez-Ojeda, Ma. Eugenia Garay-Sevilla, Arturo Figueroa

**Affiliations:** 1 Department of Medical Sciences, Division of Health Sciences, University of Guanajuato, Campus León, León CP. 37320, Guanajuato, Mexico; alan.rodriguez@ugto.mx (A.A.R.-C.); kavati75@hotmail.com (K.V.-O.); lorena.ibarra@ugto.mx (L.d.R.I.-R.); armando.gomez@ugto.mx (A.G.-O.); 2 Department of Kinesiology and Sport Management, Texas Tech University, Lubbock, TX 79409, USA

**Keywords:** L-citrulline, HIIT, MASLD, adolescents, arterial stiffness

## Abstract

Background: Metabolic-dysfunction-associated steatotic liver disease (MASLD) and obesity contribute to vascular dysfunction through oxidative stress, heightening cardiovascular risk. Oral supplementation with L-citrulline (L-cit), a precursor of L-arginine (L-arg) and nitric oxide, and high-intensity interval training (HIIT) may improve vascular function and cardiometabolic health. Objectives: This study aimed to evaluate the combined effects of L-cit supplementation and HIIT on arterial stiffness, body composition, glucose metabolism, lipid profile, and blood pressure (BP) in adolescents with MASLD and obesity. Methods: In this double-blind, placebo-controlled, randomized clinical trial (ClinicalTrials.gov (NCT05778266), 44 adolescents (15–19 years) with MASLD and obesity were assigned to HIIT + L-cit (n = 14), HIIT + placebo (n = 14), or L-cit (n = 15) for 12 weeks. HIIT sessions (85% and 60% peak heart rate during intense and recovery periods) occurred thrice weekly. Training volume progressively increased, and participants performed 20 min of HITT per session in the last 8 weeks. Results: Outcomes included pulse wave velocity (PWV), augmentation index (Aix75), VO2peak, body composition, BP, glucose and lipid profiles, and hepatic steatosis. Compared to L-cit, HIIT + L-cit improved non-high-density lipoprotein cholesterol (*p* = 0.04), very-low-density lipoprotein cholesterol (*p* = 0.01), triglycerides (*p* = 0.02), and VO2peak (*p* = 0.001). No significant between-group changes were found in PWV, AIx75, hepatic steatosis, and body composition. HIIT + placebo improved VO2peak (*p* = 0.002), and L-cit decreased the degree of steatosis (*p* = 0.038). Conclusions: HIIT + L-cit supplementation enhanced lipid profile and cardiorespiratory fitness, while HIIT + placebo improved cardiorespiratory capacity, and L-cit alone decreased hepatic steatosis. Thus, L-cit could be an adjuvant strategy to manage obesity-related MASLD in adolescents.

## 1. Introduction

Obesity is a condition with excessive accumulation of adipose tissue due to an imbalance between caloric intake and expenditure [1]. Approximately 160 million children and adolescents worldwide were classified as obese in 2022, and the obesity prevalence continues to increase dramatically [2]. In Mexico, overweight and obesity affect between 35–40% of adolescents aged 12 to 19 years, with a lower prevalence in females than in males [3]. Obesity is associated with cardiometabolic risk factors such as hypertension, dyslipidemia, hyperglycemia, and liver steatosis that increase the risk of cardiovascular disease [4,5].

Although body mass index (BMI) relates to obesity, it is based on body weight and does not distinguish between fat and lean mass [6]. Therefore, the use of imaging or impedance technology is recommended to evaluate body composition. Bioelectrical impedance (BIA) is based on the resistance of body fluids, cells, and tissues when an alternating current is passed through them. BIA provides body composition measurements such as fat mass and lean mass [7].

In metabolic-dysfunction-associated steatotic liver disease (MASLD), previously termed non-alcoholic fatty liver disease (NAFLD), obesity and other cardiometabolic risk factors are tightly linked [8]. Excessive intake of carbohydrates and fatty acids increases visceral adipose tissue, enhancing the mobilization of free fatty acids to the liver. This process promotes inflammation, low adiponectin levels, and insulin resistance, contributing to the development of hepatic steatosis [9]. The resulting imbalance drives the hepatic storage of fatty acids as triglyceride droplets [10]. Additionally, these mechanisms enhance fatty acid oxidation, generating reactive oxygen species (ROS) and oxidative stress. This oxidative stress reduces endothelial nitric oxide (NO) production, leading to endothelial dysfunction [11].

Endothelial dysfunction is characterized by reduced NO bioavailability and impaired vasodilation [12]. NO production is largely dependent on L-arg availability, as it is the only substrate for endothelial NO synthase (eNOS) [13]. However, increased ROS enhances arginase activity, which competes with eNOS for L-arg, diverting L-arg to urea and ornithine rather than to NO production by eNOS [14]. Additionally, increased asymmetric dimethylarginine (ADMA) competes with L-arg for eNOS binding, inhibiting NO synthesis and contributing to hypercholesterolemia, atherosclerosis, and hypertension [15].

Arterial stiffness, a hallmark of vascular dysfunction, can be non-invasively assessed by carotid–femoral pulse wave velocity (PWV), a reliable biomarker of central arterial stiffness. The augmentation index (AIx), a measure of pressure wave reflection, is normalized to a heart rate (HR) of 75 beats per minute (AIx75) to account for its inverse correlation with HR [16].

Although the amino acid L-arg is the precursor of NO [14], long-term supplementation efficacy becomes limited by stimulation of arginase activity leading to reduced L-arg availability for NO synthesis [17]. L-citrulline (L-cit) is the precursor of L-arg and has shown to efficiently increase plasma L-arg levels and endothelial-mediated vasodilation without adverse effects [17,18]. Evidence shows that L-cit decreases liver steatosis [19], low-density lipoprotein cholesterol (LDL-C) levels [20], blood pressure (BP), and peripheral arterial stiffness in middle-aged adults [21,22].

A study evaluated the effects of oral L-cit supplementation on liver function and steatosis in adolescents with abdominal obesity and NAFLD, marking the first investigation of its kind in this population. L-cit for 8 weeks significantly reduced LDL-C and levels of liver steatosis, particularly in those with mild and severe NAFLD. This reduction was attributed to enhanced L-arg production and inhibition of lipogenesis in hepatocytes [20].

High-intensity interval training (HIIT) consists of exercise bouts at 85–95% of maximum HR interspersed with low-intensity periods [23]. The main appeal of HIIT is that this type of training can be completed in a short period of time compared to traditional continuous aerobic training, resulting in comparable physiological adaptations [24]. Some clinical trials have demonstrated that HIIT improves arterial function and decreases BMI and body fat mass (absolute and percentage) [25,26].

HIIT has been increasingly studied as a potential therapeutic approach for metabolic health, particularly in populations with obesity and MASLD. Although limited data exist on HIIT’s effects in adolescents, De Lira et al. evaluated its efficacy in adolescents with obesity and NAFLD, aiming to address concerns about potential oxidative stress and hepatic damage. After 12 weeks of HIIT, reductions in BMI and improvements in lipid profile were observed [27], supporting the intervention’s safety and potential benefit in young populations. Studies in adults further reinforce the impact of HIIT on body composition, lipid profiles and metabolic markers. For instance, 8–12 weeks of HIIT improved fat mass percentage, BMI, and high-density lipoprotein cholesterol (HDL-C) [28] in adults with NAFLD [28,29].

There are few studies that have combined HIIT with L-cit supplementation. In a study by Buckinx et al., the effects of a 12-week HIIT program combined with L-cit supplementation (10 g/day) were compared to HIIT with a placebo in older adults with obesity. The HIIT and L-cit group experienced reductions in total fat mass and total cholesterol (total-C), while these effects were not observed in the HIIT and placebo group [30]. Previous studies using strength training and L-cit supplementation have demonstrated synergistic benefits on vascular health. Improvements were observed in arterial stiffness and endothelial function, primarily through upregulation of NO synthesis [21,22,31]. Studies indicated that chronic L-cit supplementation, particularly when combined with exercise training, can reduce peripheral arterial stiffening and aortic BP [21,22] or enhance muscle mass and function [31].

Given the limited research on interventions targeting cardiometabolic health in obese adolescents with MASLD, this study aimed to test the hypothesis that L-cit supplementation combined with HIIT would improve liver steatosis, body composition, glucose metabolism, lipid profile, arterial stiffness, and BP compared to each intervention alone in adolescents with MASLD and obesity. This approach seeks to offer insights into the effectiveness of these interventions on cardiovascular health in a young population at high cardiometabolic risk.

## 2. Materials and Methods

### 2.1. Subjects

This study enrolled adolescents aged 15 to 19 years from high schools of León, Guanajuato, diagnosed with liver steatosis and obesity (BMI > 30 kg/m^2^). Eligible participants were those classified at Tanner stage IV or V, who reported a sedentary lifestyle, were non-smokers, and had no history of medication, dietary supplement use, or structured exercise programs for at least six months prior to the study. Exclusion criteria included intolerance to L-cit supplementation, adherence below 80% to the prescribed protocols, or a diagnosis of chronic diseases. Participant information, including tobacco use, medication or supplement intake, Tanner stage, and history of chronic conditions, was collected through a validated questionnaire. This study was approved by the Ethics Committee for Research of the Universidad de Guanajuato and was registered in ClinicalTrials.gov (NCT05778266). All participants and their guardians signed an informed consent form after the research procedures were explained to them.

### 2.2. Study Design

This study employed a randomized, double-blind (participants and investigator), parallel-group design conducted from March to December 2023. Participants were randomly assigned using a computer-generated sequence by an independent investigator to one of three groups: HIIT with L-cit supplementation (HIIT + L-cit, n = 14), HIIT with placebo (HIIT + placebo, n = 14), or L-cit supplementation alone (L-cit, n = 15) for a 12-week intervention. Baseline (week 0) and final (week 12) measurements were performed to evaluate the effects of the intervention across groups. For the calculation of sample size, the changes in arterial stiffness (PWV) in response to HIIT in 10 adolescents after the intervention were considered [32], with a power of 80% and a level of α = 0.05. A total of 12 participants per group were calculated, and an increase of 20% was considered for possible dropouts by selecting 15 participants per group.

Anthropometric measurements, including height, weight, and waist circumference, were taken with participants barefoot and wearing light clothing. Height was recorded to the nearest 0.1 cm using a stadiometer (Seca 406), while weight was measured to the nearest 0.1 kg with an electronic scale (Tanita HD 357 Scale). Waist circumference was assessed to the nearest 0.1 cm with a Lufkin tape at the midpoint between the lower costal margin and the anterior superior iliac crest. BMI was subsequently calculated as weight (kg) divided by height squared (m^2^), and obesity was defined as a BMI-for-age exceeding 2 standard deviations above the WHO median [1].

The degree of liver steatosis was evaluated using an Acuson X150-03 ultrasound system with a 2–5 MHz multifrequency convex transducer. (Siemens Medical Solutions, Inc. Mountain View, CA, USA) Fat accumulation and the dimensions of the liver lobes were assessed by two trained specialists. The diagnosis was established according to the following criteria: (1) increased echogenicity compared to the renal cortex; (2) identification of non-compromised regions, defined as areas of the liver free from fatty infiltration; (3) clear visualization of the walls of the portal vessels and diaphragm; and (4) sound attenuation. Steatosis was classified as mild, moderate, or severe [33].

### 2.3. Biochemical Analyses

A venous blood sample was obtained after 12 h of overnight fasting. Serum was processed and centrifuged for 10 min at 4 °C the same day. Then, aliquots of 400 µL were frozen at −80 °C until assay. Glucose was determined by the glucose oxidase method (GOD-PAD Lakeside). Lipid profile (total-C, HDL-C, VLDL-C, and triglycerides) was measured by a modified Huang method of the Spinreact brand. LDL-C was calculated using the Friedewald formula, expressed as LDL-C = Total-C − HDL-C − (Triglycerides/5). Insuline and L-arg were determined by ELISA kits from MyBioSource (Southern California, San Diego, CA, USA). The homeostasis model assessment of insulin resistance HOMA-IR index was calculated as (glucose × insulin)/22.5. In females, the measurements were performed in the first half of their ovulatory cycle.

### 2.4. Peak Oxygen Consumption (VO2peak)

Participants performed an exercise stress test on a cycle ergometer (Monark, Ergomedic 828 E) that was calibrated before each test. The test consisted of multistage incremental effort with a progressive increase in the load in each stage. Briefly, the test consisted of a period of familiarization with rhythmic pedaling for a 5 min warm up period. Thereafter, participants rhythmically pedaled at 60 rpm and at a 1.5 kp load for 2 min, which was gradually increased by 0.5 kp every 2 min until the test was completed. Both the basal heart rate and HR peak were recorded every minute with a HR monitor (Polar OH1, Polar Electro, Kempele, Finland). The test ended when they reached exhaustion or if the participants did not keep with the cadence of pedaling. The leg-ergometer equation was used to estimate the VO2peak [34]. VO2peak (mL/kg/min) = 1.8 [work rate (kg m/min)/body mass (kg)] + 7. VO2peak was used as an index of cardiorespiratory fitness. To rule out contraindications for exercising, a 12-lead electrocardiogram (ECG) was obtained before the test (Combo Resting 12-Lead ECG. 4.0 Premier, DM Software, Stateline, NV, USA).

### 2.5. Blood Pressure and Arterial Function

BP, PWV, AIx75, and measurements were conducted in a quiet, temperature-controlled room with regulated lighting and minimal ambient noise. Following 10 min of seated rest, brachial BP was measured on the non-dominant arm using an automatic device (OMRON Healthcare Inc., Shanghai, China). Three readings were taken, each separated by a 2 min interval, and the average value was used for analysis.

Arterial function parameters were assessed using a validated Mobil-O-Graph device (IEM GmbH, Stolberg, Germany). This is a validated, non-invasive device that uses the oscillometric method to perform BP, PWV, and AIx75 measurements through a single-point brachial technique. Measurements were made in a seated position after at least 10 min of rest. Measurements were repeated after a 5 min rest. If the PWV values differed by more than 0.5 m/s between readings, a third measurement was performed. The final PWV value was calculated as the average of two measurements.

### 2.6. Body Composition

Body composition (total body fat mass, body fat mass percentage, skeletal muscle weight, visceral fat area) was measured using bioelectrical impedance analysis (model s10, InBody). Measurements were taken with participants seated and after 10 min of rest.

### 2.7. Dietary Assessment

Dietary intake was assessed using 24 h dietary recalls conducted on three non-consecutive days, including one weekend day, to account for variations in daily intake. Trained researchers guided participants through the recall process to ensure accuracy, with portion sizes estimated using standardized food models and portion guides. Nutrient analysis, including total energy (kcal), macronutrients (carbohydrates, protein, fats), and micronutrients, was performed using the Food Processor Nutrition Analysis Software 11.6.522 (ESHA Research, Salem, OR, USA).

### 2.8. Interventions

Participants in the HIIT + L-cit and HIIT + placebo groups completed supervised training on a stationary bike 3 times a week (Monday, Wednesday, and Friday) for 12 weeks. Participants were instructed to cycle at a workload of 85% and 60% of their HR peak during the intense and recovery periods, respectively. HR monitors (Polar OH1, Polar Electro, Finlandia) were used to achieve the target HR. Each exercise session consisted of a 5 min warm-up and a 5 min cool-down. The training volume was systematically increased by augmenting the number of sets and exercise duration. During the first two weeks, participants completed 6 sets, each set consisting of 6 min of intense period followed by 6 min of recovery per session. In weeks three and four, they progressed to 8 sets, consisting of 8 min of intense work and 8 min of recovery per session. From weeks five to twelve, participants performed 10 sets, consisting of 10 min of intense effort followed by 10 min of recovery per session. The progressive training model employed in this study was adapted from a previous investigation in participants with type 2 diabetes and obesity [35]. Given that our study population consisted of sedentary individuals, we chose to implement a progressive approach to facilitate their adaptation to the exercise regimen and improve their overall acceptance of the program [35].

The L-cit and placebo capsules were similar in size and color and provided weekly in labeled bottles. Participants in the HIIT + L-cit and L-cit groups consumed 6.6 g of L-cit per day, administered as 737 mg capsules (Abastecedora de productos naturales—Pronat Prowinner^®^). Each participant took 5 capsules (3.69 g) in the morning, 30 min before the first meal, and 4 capsules (2.95 g) in the evening before dinner. Similarly, the HIIT + placebo group ingested carboxymethylcellulose capsules under the same dosing instructions. The selected L-cit dosage and timing were based on previous studies that demonstrated reductions in BP and arterial stiffness, along with improvements in body composition [20,36,37,38]. Text messages were sent to participants in the morning and night reminding them to take the capsules. Follow-up and adherence were assessed with Morinsky–Green test and capsule counting to ensure at least 80% compliance.

### 2.9. Ethics Approval

This study was approved by the Ethics Committee for Research of the University of Guanajuato (CEPIUG P68-2022) and registered in ClinicalTrials.gov (NCT05778266). After explaining the research procedure, the participants and their parents signed and informed consent form.

### 2.10. Statistical Analysis

The Shapiro–Wilk test was run to assess the normality of the data distribution. The data that presented a normal distribution were expressed as means and standard deviations, while data with a non-parametric distribution were expressed as medians and interquartile ranges. A comparison between the basal and final means was performed using Student’s *t* test for paired samples and Wilcoxon’s test to compare medians. The differences in the means or medians between the final and initial measurements were used to compare groups; the one-way ANOVA test was performed for means, and the Kruskal–Wallis test was performed for medians. The Bonferroni post hoc test was applied to variables with a *p*-value < 0.05 to identify significant differences between specific groups. The SPSS software version 25 was employed for data analysis. Significant differences were considered with a *p* value < 0.05 at a confidence level of 95%.

## 3. Results

**Figure 1:**
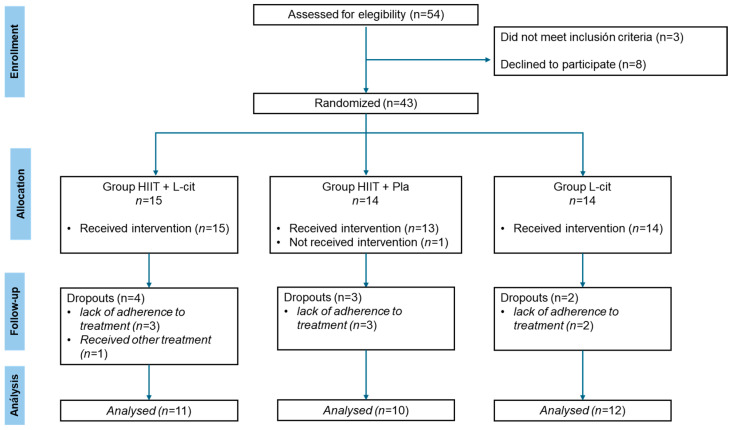
CONSORT flow diagram.

A flow diagram depicting the progress of the participants in the trial is shown in Figure 1. Of the 54 adolescents evaluated, only 43 (33 female and 9 male) were randomized and allocated in the intervention groups. Compliance to L-cit in the HIIT + L-cit and L-cit groups was 89.4% and 91.5%, respectively. Compliance to HIIT was 93.1% and 91% in the HIIT + L-cit and HIIT + placebo groups, respectively. There were no adverse effects to the supplements or injury related to the exercise training.

Baseline characteristics of the participants are presented in Table 1. There were no significant differences between the groups in terms of the baseline variables. The participants had normal BP (n = 11), elevated BP (n= 13), and hypertension (n= 9).

As shown in Table 2, significant within-group improvements were observed in the HIIT + L-cit group, including reductions in BMI (*p* = 0.03), fat mass (*p* = 0.02), body fat mass (*p* = 0.02), visceral fat area (*p* = 0.02), VLDL-C (*p* = 0.007), triglycerides (*p* = 0.01), systolic blood pressure (SBP) (*p* = 0.01), and PWV (*p* < 0.001); significant increase were also observed in terms of VO2peak in the HIIT + L-cit and HIIT + placebo group, as well as an increase in total-C and non-HDL-C in the last group. However, no statistically significant differences were found in the other variables in the study, as shown in Table 2.

As observed in Table 3, the L-cit group exhibited a significant improvement in the steatosis grade following the intervention (*p* = 0.038), while no significant changes were detected in the HIIT + L-cit and HIIT + placebo groups. Specifically, of the three patients with mild steatosis, all remained unchanged. Among the six patients with moderate steatosis, four improved to mild steatosis, while two remained at the same grade. Notably, all three patients with severe steatosis improved to moderate steatosis. In contrast, no significant changes in steatosis grade were detected in the HIIT + L-cit and HIIT + placebo groups.

## 4. Discussion

This study is the first to assess both the individual and combined effects of L-cit supplementation and HIIT on body composition, hepatic steatosis, lipid and glucose metabolism, and vascular function in adolescents with MASLD. The key findings suggest that L-cit combined with HIIT significantly improves VO2peak, VLDL-C, non-HDL-C, and triglycerides. HIIT alone enhanced VO2peak, while L-cit alone reduced hepatic steatosis severity. In addition, significant improvements were also observed within individual groups. The HIIT + L-cit group demonstrated reductions in fat mass, body fat percentage, visceral fat area, SBP, PWV, and AIx75. Meanwhile, the L-cit group exhibited a notable increase in HDL-C. These findings highlight the potential benefits of L-cit combined with HIIT or alone on cardiometabolic risk factors in obesity-related MASLD.

There were significant reductions in VLDL-C, non-HDL-C, and triglycerides following HIIT + L-cit. Moreover, there were no notable changes in LDL-C or HDL-C following the intervention. In contrast, Meng et al. observed a decrease in LDL-C after 12 weeks of HIIT in preadolescents, yet they found no significant changes in triglyceride levels [39]. Our findings on total-C and LDL-C in the L-cit group diverge from those of Tovar-Villegas et al., who reported reductions in LDL-C, which may be attributable to the larger sample size in the previous study [20]. The reduction in triglycerides following HIIT + L-cit aligns with findings from a previous study after 12 weeks of HIIT + L-cit supplementation in obese older patients [40]. In disagreement with our findings, L-cit has been shown to be ineffective in reducing triglycerides in obese diabetic mice [41]. Despite the fact that L-cit reduced serum triglycerides in the patients with T2D, the decrease was not significant compared to the placebo [42]. Thus, the decrease in LDL-C and triglyceride levels following HIIT + L-cit may be mainly attributed to L-cit and potentiated by HIIT. The beneficial effect of L-cit on VLDL-C and triglycerides may be related to reduced hepatic lipogenesis via a NO-mediated mechanism [43].

In terms of body composition, participants in the HIIT + L-cit group exhibited reductions in fat mass, body fat percentage, and visceral fat area, which likely contributed to their overall weight loss. These results were not observed in the groups receiving either HIIT or L-cit supplementation alone, and no differences were found between groups either. Previous studies have shown that HIIT over 12 to 16 weeks can effectively reduce fat mass and body fat percentage while increasing lean mass in children, adolescents, and young adults with obesity [27,39,44,45,46]. While these studies utilized dual-energy X-ray absorptiometry for body composition assessment, our study employed bioelectrical impedance analysis, which can yield different results based on hydration status. For instance, Babu et al. assessed body composition through impedance in adults with obesity and MAFLD and found no significant changes after 12 weeks of HIIT [47]. Studies comparing running and cycling have demonstrated that running is more effective in reducing both total and visceral fat mass. Moreover, high-intensity exercise, particularly at intensities exceeding 90% of the HR peak, appears to be more effective in decreasing overall body adiposity, while lower intensities are more successful in reducing abdominal and visceral fat mass. In our protocol, participants performed the training using a cycling modality at an intensity of 85% HR peak [48]. This difference in exercise modality and intensity could potentially explain the absence of significant reductions in fat mass observed in our study.

Few studies have specifically investigated the impact of L-cit on body composition. Bouillanne et al. reported that a 3-week supplementation of L-cit (10 g/day) resulted in decreased fat mass in malnourished elderly women, attributed to enhanced fatty acid release from visceral adipose tissue [49]. Additionally, a study demonstrated that L-cit for 12 weeks reduced intra-abdominal fat mass in older rats, likely due to increased fatty acid release from visceral adipose tissue and decreased glyceroneogenesis [50]. To date, no studies have examined changes in body composition following L-cit supplementation in adolescents. This lack of evidence makes it difficult to directly compare our findings, particularly in the L-cit group, where no statistically significant changes were observed. However, it is noteworthy that the HIIT + L-cit group achieved statistically significant reductions in total fat mass, fat percentage, and visceral fat area. This suggests that L-cit may enhance the lipolytic effects of HIIT in this population.

We observed a significant reduction in PWV, SBP, and pulse pressure in the HIIT + L-cit group, whereas the AIx75 demonstrated only a marginal decrease after 12 weeks. However, no statistically significant differences were observed between groups. A reduction in brachial-ankle PWV (baPWV) was observed after 12 weeks of HIIT in preadolescents with obesity [25]. In contrast, PWV significantly decreased in individuals at high cardiovascular risk following HIIT [51]. Previous studies have highlighted the beneficial effects of L-cit on arterial stiffness in adults. Ochiai et al. observed a decrease in baPWV after just seven days of L-cit supplementation in healthy adult men [52]. Importantly, baPWV includes central and peripheral PWV, and previous studies have shown decreases in peripheral but not in central PWV [22]. Despite these promising findings, our study did not detect significant changes in central PWV in the L-cit group.

This discrepancy may be attributed to differences in study populations and methodology. Previous studies have shown the inefficiency of L-cit to reduce PWV and SBP in normotensive and apparently healthy adults, including young overweight men [53]. Additionally, prior studies have employed arterial tonometry, which directly measures PWV and AIx75, while our study utilized an oscillometric device. Although oscillometry is a validated, non-invasive approach and correlates well with invasive methods [54,55], it provides an indirect assessment of arterial stiffness, which could partly explain the lack of observed changes.

The adolescents in our study presented elevated cardiometabolic risk due to various comorbidities, including elevated SBP. While some participants exhibited normal BP, others presented with elevated BP or hypertension, suggesting that the inclusion of normotensive participants may have negatively influenced our results. Previous studies have demonstrated that L-cit supplementation is more effective in populations with elevated BP and hypertension, likely due to impaired endothelial dysfunction [53]. This difference in baseline vascular tone may explain the variability in our findings and highlights the need for future studies to stratify participants based on their endothelial function assessed as brachial artery flow-mediated dilation.

Physical activity is a key non-pharmacological intervention for patients with MASLD, as it enhances fatty acid oxidation and reduces hepatic fat content [11]. However, in both HIIT groups in our study, no significant changes in hepatic steatosis were observed, which contrasts with findings by Hallsworth et al., who reported a reduction in intrahepatic fat percentage after 12 weeks of HIIT, which involved 30–40 min sessions thrice weekly [28]. A potential explanation for the superior results in intrahepatic fat reduction observed in the previous study could be the shorter session duration in the present study, which was 20 min per session between weeks 5–12. Additionally, their study quantified intrahepatic lipids using magnetic resonance imaging, a highly sensitive method, whereas we employed ultrasonography, which has a lower sensitivity for detecting subtle changes in liver fat content. These methodological differences may partly account for the disparity in the observed outcomes. The duration of physical intervention could yield positive effects even over short periods; however, the lack of dietary regimen in our protocol may account for the absence of steatosis improvement, as the combination of exercise and dietary modification has been shown to amplify exercise effects [56]. Our findings in the L-cit group align with those of Tovar-Villegas et al., where a reduction in steatosis was observed following 8 weeks of L-cit supplementation [20].

A significant limitation of our study is the small sample size. Although it was calculated to detect changes in PWV, a larger number of participants with elevated BP and hypertension may be necessary to achieve statistical significance in the primary variables of interest. Losses during follow-up may also have influenced our ability to obtain conclusive results in the intervention groups. Most of the participants were females, which may have positively affected the results, as previous studies have shown that L-cit exhibits pronounced effects in females in animals and humans [18,21,22,43]. To strengthen the validity and generalizability of our findings, future research with larger populations is essential. Conducting large-scale studies will not only support the current results but also help identify potential interindividual differences in MASLD physiopathology.

The study population presents a high cardiometabolic risk, and the three intervention groups received treatments aligned with existing evidence regarding the benefits of both L-cit and HIIT. Although the absence of a control group receiving a placebo and no HIIT may be viewed as a limitation, including such a group could raise ethical questions.

## 5. Conclusions

In adolescents with MASLD, a 12-week intervention with HIIT and L-cit supplementation resulted in improved cardiorespiratory capacity and lipid profile. These findings suggest a synergistic effect that may help reduce metabolic disease risk. In addition, HIIT alone improved cardiorespiratory capacity, and L-cit alone decreased the degree of steatosis. Therefore, further studies are necessary to investigate the impact of combined physical activity with L-cit in populations with greater cardiometabolic risk.

## Figures and Tables

**Figure 2:**
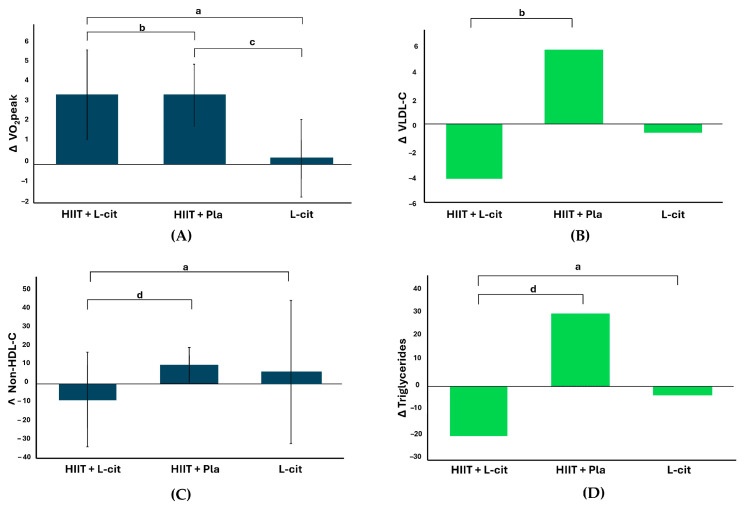
NS: not significant; VO2peak: peak oxygen consumption; VLDL: very-low-density lipoprotein; HIIT: high-intensity interval training; L-cit: L-citrulline; Pla: placebo. Absolute changes in (**A**) VO2peak, (**B**) VLDL-C, (**C**) non-HDL-C, and (**D**) triglycerides after 12 weeks of intervention. Analysis performed with one-way ANOVA (blue) or the Kruskal–Wallis test (green) according to normality of data. Post hoc comparisons were conducted using the Bonferroni test: a: *p* < 0.05 vs. L-cit; b: *p* < 0.01 vs HIIT + placebo; c: *p* < 0.01 vs. L-cit; d: *p* < 0.05 vs. HIIT + placebo.

**Table 1:** Baseline characteristics of the participants in the 3 intervention groups.

	HIIT + L-cit (*n* = 15)		HIIT + Placebo (*n* = 14)		L-cit (*n* = 14)		*p*
Age (y)	16 ± 0.9		16.1 ± 0.9		16.3 ± 0.7		0.92 ^#^
Male/Female (n)	2/13		2/12		6/8		
Anthropometry							
Weight (kg)	86 ± 8.6		89.4 ± 13.7		97.9 ± 20.1		0.09 ^#^
Height (m)	1.62 ± 0.05		1.64 ± 0.1		1.67 ± 0.1		0.35 ^#^
BMI (kg/m^2^)	32.7 (28.8, 35.3)		31.75 (29.7, 4)		32.6 (29.2, 48.6)		0.78 ^†^
Abdominal Circumference (cm)	102 ± 6		105 ± 9.8		110 ± 12.9		0.11 ^#^
Body Composition							
Fat Mass (kg)	37.9 (30.4, 45.1)		37.4 (31.6, 60.9)		38.8 (24.7, 63.7)		0.86 ^†^
Body Fat Mass (%)	44.5 ± 4.1		44.4 ± 6.3		43 ± 7.3		0.74 ^#^
Visceral Fat Area (cm^2^)	178 ± 18.6		184 ± 39.6		185 ± 42		0.81 ^#^
Muscle Mass	24.7 (22.7, 37.2)		26.7 (22.5, 43.9)		29.2 (21.7, 47)		0.15 ^†^
Glucose Control							
Glucose (mg/dL)	93.4 ± 7.5		94.2 ± 7.8		96.4 ± 13.4		0.69 ^#^
Insulin (µU/mL)	49 ± 18.4		36.9 ± 19.8		45.7 ± 28.3		0.34 ^#^
HOMA-IR	10.4 (4.66, 25.6)		7.2 (1.2, 21.4)		8.6 ± 32.1		0.23 ^†^
HbA1C (%)	4.2 (4.2, 4.8)		4.2 (4.2, 5.4)		4.2 ± 0.9		0.61 ^†^
Lipid Profile							
Total-C (mg/dL)	162.4 ± 30.3		150.1 ± 19.7		167.6 ± 34.3		0.26 ^#^
HDL-C (mg/dL)	45.1 ± 7.9		45.6 ± 12.6		43.1 ± 5.9		0.72 ^#^
Non-HDL-C (mg/dL)	117.3 ± 27.6		104.5 ± 15.2		124.5 ± 33.8		0.14 ^#^
LDL-C (mg/dL)	89.5 ± 24.1		85.9 ± 14.4		98.9 ± 28.1		0.30 ^#^
VLDL-C (mg/dL)	27.9 ± 13.1		18.5 ± 6.3		25.6 ± 10.9		0.06 ^#^
Triglyceride (mg/dL)	139.7 ± 65.8		91.1 ± 32.1		128.8 ± 54.9		0.06 ^#^
Atherogenic Index	3.7 ± 0.8		3.4 ± 0.7		3.9 ± 0.8		0.20 ^#^
Vascular Function							
PWV (m/s)	4.86 ± 0.3		4.86 ± 0.3		4.92 ± 0.5		0.89 ^#^
AIx75 (%)	27.2 ± 7.2		29.7 ± 8.8		28.7 ± 6.4		0.67 ^#^
Arginine (µU/dL)	64.2 (0.65, 203)		54.2 (0.3, 263)		55.4 (0.70, 130.5)		0.38 ^†^
Blood Pressure							
Systolic (mmHg)	120 ± 8		120 ± 11		122 ± 14		0.91 ^#^
Diastolic (mmHg)	67 ± 6		66 ± 5		69 ± 9		0.62 ^#^
Median (mmHg)	85 ± 6		84 ± 6		86 ± 10		0.71 ^#^
Systolic Central (mmHg)	118 ± 9		115 ± 6		119 ± 12		0.39 ^#^
Diastolic Central (mmHg)	68 ± 6		69 ± 8		70 ± 10		0.89 ^#^
Pulse Pressure (mmHg)	53 ± 7		54 ± 10		53 ± 12		0.93 ^#^
Cardiorespiratory Fitness							
VO2peak (mL/kg/min)	29.3 ± 2.8		28.3 ± 3.9		27.8 ± 4.5		0.55 ^#^
Heart Rate (bpm)	78.1 ± 11.7		80.1 ± 11.9		80.1 ± 14.7		0.88 ^#^

BMI: body mass index; VO2peak: peak oxygen consumption; HOMA IR: Homeostasis Assessment Model of Insulin Resistance; HbA1c: glycated hemoglobin C: cholesterol; HDL: high-density lipoprotein; LDL: low-density lipoprotein; VLDL: very-low-density lipoprotein; PWV: pulse wave velocity; AIx75: augmentation index corrected to 75 beats per minute; bpm: beats per minute. Data are expressed as mean ± SD or median (range) according to normality. Statistical significance *p* < 0.05. Analyses were performed using one-way ANOVA (**^#^**) or the Kruskal–Wallis test (**^†^**).

**Table 2:** Parameters at baseline and final and changes after interventions.

	HIIT + L-cit				HIIT + Pla				L-cit				
	(*n* = 11)				(*n* = 10)				(*n* = 12)				
	Baseline	Final	*p*-Value Within Group	∆	Baseline	Final	*p*-Value Within Group	∆	Baseline	Final	*p*-Value Within Group	∆	*p*-Value Between Group
Age	16 ± 0.89				16.10 ± 0.88				16.25 ± 0.75				
Male/Female	2/9				1/9				5/7				
Antrhopometry													
Weight (kg)	86.3 ± 9.9	85.3 ± 10.2	0.06	−1.1 ± 1.7	89.9 ± 15.9	89.5 ± 15.3	0.69	−0.4 ± 0.9	92.2 ± 15.7	92.4 ± 15	0.63	0.2 ± 1.3	0.23 ^#^
Height (m)	1.6 ± 0.1	1.6 ± 0.1	0.34	0	1.6 (1.49, 1.92)	1.6 (1.49, 1.92)	1	0	1.7 ± 0.1	1.7 ± 0.1	0.36	0	0.36 ^#^
Abdominal Circumference (cm)	101 ± 6.3	102 ± 6.7	0.68	−0.5 ± 3.8	106 ± 11.1	105 ± 8.5	0.61	−0.8 ± 5.2	107 ± 12.4	108 ± 11.7	0.39	0.4 ± 4.6	0.75 ^#^
BMI (kg/m^2^)	32.6 ± 2.2	32.2 ± 2.4	0.03	−0.5 ± 0.6	33.6 ± 3.9	33.4 ± 3.4	0.69	−0.1 ± 1.12	34.6 ± 5.5	34.8 ± 5.5	0.57	0.2 ± 0.4	0.15 ^#^
Body Composition													
Fat Mass (kg)	37.8 ± 3.8	36 ± 4	0.02	−1.8 ± 2.2	40.8 ± 9.6	39.9 ± 8.2	0.47	−0.9 ± 3.8	41.9 ± 10.8	41.7 ± 10.7	0.75	−0.2 ± 2.1	0.36 ^#^
Body Fat Mass (%)	43.9 ± 3.4	42.3 ± 4.1	0.02	−1.6 ± 1.9	45.4 ± 6.32	44 ± 5.94	0.26	−1.4 ± 3.8	42.9 ± 6.48	42.4 ± 6.94	0.49	−0.5 ± 2.4	0.60 ^#^
Visceral Fat Area (cm^2^)	179 ± 17.15	168 ± 21.84	0.02	−10.7 ± 13.9	192 ± 37.4	183 ± 38.2	0.18	−9.1 ± 17.4	187 ± 37.3	185 ± 37.3	0.94	−1.5 ± 11.4	0.21 ^#^
Muscle Mass	24.7 (23, 37.20)	26.1 (23.1, 38.6)	0.2	1.4 (−2.2, 4.7)	25.6 (22.9, 43.9)	25.7 (20.3, 44.9)	0.26	0.1 (−2.6, 2.0)	29.2 (21.7, 47)	30.3 (20.3, 47.7)	0.42	1.1 (−1.8, 2.6)	0.83 ^†^
Cardiorespiratory Fitness													
VO2peak (ml/kg/min)	29.8 ± 3.1	32.6 ± 3.4	0.002	3.4 ± 2.2 ^ab^	27.6 ± 3.8	30.9 ± 3.8	<0.0001	3.4 ± 1.5 ^c^	26.8 ± 4.3	27.1 ± 4.9	0.6	0.3 ± 1.9	0.001 *^#^
Heart Rate (lpm)	77.5 ± 12.1	75.8 ± 9.1	0.59	−1.5 ± 9.7	79 ± 11.6	75.5 ± 10.7	0.37	−3.3 ± 11.6	82.7 ± 12.4	82.5 ± 14.1	0.68	−0.3 ± 9.4	0.76 ^#^
Glucose Control													
Glucose (mg/dL)	94.1 ± 7.3	96.1 ± 4.8	0.5	2 ± 9.6	93.4 ± 8	99.8 ± 9.1	0.06	6.4 ± 9.82	94.4 ± 9.8	104 ± 9.9	0.006	10.91 ± 8.43	0.08 ^#^
Insulin (µU/mL)	48.9 ± 20.8	38.1 ± 13.1	0.11	−10.7 ± 20.6 ^a^	33.9 ± 17.6	33 ± 13.1	0.83	−0.90 ± 13.11	46.6 ± 31	58.3 ± 37.8	0.52	11.70 ± 26.21	0.05 *^#^
HOMA-IR	11.6 ± 5.7	9.1 ± 3.2	0.18	−2.5 ± 5.9 ^a^	7.8 ± 3.9	8.1 ± 3.2	0.75	0.35 ± 3.48	12 ± 9.7	16.6 ± 13.4	0.28	4.56 ± 7.88	0.03 *^#^
HbA1C (%)	4.2 (4.2, 4.8)	4.2 (4.2, 5.4)	0.68	0 (−0.6, 1.2)	4.2 (4.2, 4.9)	4.2 (4.2, 4.7)	0.71	0 (−0.20, 0.50)	4.2 (4.2, 4.5)	4.2 (4.2, 5.8)	0.71	0 (−0.30, 1.50)	0.56 ^†^
Lipid Profile													
Total-C (mg/dL)	169 ± 31.1	163.6 ± 29.9	0.45	−5.5 ± 23.1	149.3 ± 12.6	161.1 ± 14.2	0.008	11.8 ± 11	162.3 ± 32.6	173 ± 33.3	0.96	10.8 ± 39.1	0.28 ^#^
HDL-C (mg/dL)	45.6 ± 8.7	48.5 ± 10.5	0.26	2.8 ± 7.9	43.2 ± 9.1	44.9 ± 7.4	0.39	1.7 ± 6.1	42.8 ± 6.9	48.9 ± 7.3	0.01	4.4 ± 7.3	0.67 ^#^
LDL-C (mg/dL)	96.9 ± 21.8	96.4 ± 23.8	0.88	0.5 ± 12.4	87.4 ± 7.1	95 ± 11.2	0.07	7.6 ± 12	93.5 ± 23.8	99.4 ± 24.8	0.77	5.9 ± 29.7	0.62 ^#^
VLDL-C (mg/dL)	20 (12, 61)	16 (10, 38)	0.007	−4 (−5, 2) ^b^	17 (12, 36)	20.5 (15, 30)	0.11	5.5 (−19, 11)	23 (11, 52)	21.5 (12, 49)	0.29	−0.5 (−19, 38)	0.01 ^†^
Non-HDL-C (mg/dL)	123.4 ± 28.6	115.1 ± 27.1	0.29	−8.3 ± 25.1 ^ad^	106.1 ± 8	116.2 ± 11.76	0.007	10.1 ± 9.3	119.3 ± 32.4	125.6 ± 32.8	0.41	6.3 ± 38.8	0.04 *^#^
Atherogenic Index	3.8 ± 0.9	3.5 ± 0.9	0.39	−0.3 ± 98	3.5 ± 0.6	3.7 ± 0.6	0.55	0.1 ± 0.6	3.8 ± 0.8	3.8 ± 0.9	0.16	−0.1 ± 1.	0.40 ^#^
Triglyceride (mg/dL)	98 (62, 306)	82 (50, 189)	0.01	−20 (−250, 12) ^ad^	83 (59, 181)	103 (75, 150)	0.09	29.5 (−95, 55)	117 (53, 260)	108 (61, 246)	0.25	−3.5 (−96, 193)	0.02 *^†^
Blood Pressure													
Systolic (mmHg)	122 ± 7	119 ± 7	0.01	−3 ± 4	121 ± 12	117 ± 8	0.22	−4 ± 10	125 ± 14	122 ± 14	0.92	−3 ± 8	0.84 ^#^
Diastolic (mmHg)	68 ± 4	68 ± 5	0.93	0 ± 4	64 ± 5	68 ± 6	0.29	4 ± 9	72 ± 8	72 ± 10	0.79	0 ± 9	0.63 ^#^
Mean (mmHg)	86 ± 4	85 ± 5	0.33	−1 ± 4	83 ± 6	84 ± 6	0.78	1 ± 8	89 ± 9	89 ± 10	0.86	0 ± 8	0.75 ^#^
Systolic Central (mmHg)	120 ± 10	117 ± 9	0.08	−3 ± 5	115 ± 6	116 ± 9	0.16	1 ± 7	121 ± 13	120 ± 12	0.59	−1 ± 10	0.47 ^#^
Diastolic Central (mmHg)	69 ± 4	68 ± 6	0.72	−1 ± 5	68 ± 10	69 ± 6	0.64	1 ± 11	73 ± 8	74 ± 9	0.31	1 ± 9	0.64 ^#^
Pulse Pressure (mmHg)	55 ± 8	51 ± 7	0.03	−4 ± 5	57 ± 11	50 ± 6	0.03	−7 ± 8	53 ± 13	52 ± 12	0.42	−1 ± 9	0.25 ^#^
Vascular Function													
PWV (m/s)	4.95 ± 0.2	4.76 ± 0.2	<0.001	−0.2 ± 0.1	4.9 ± 0.3	4.74 ± 0.3	0.06	−0.2 ± 0.2	5.01 ± 0.5	4.92 ± 0.5	0.73	−0.1 ± 0.3	0.57 ^#^
AIx75 (%)	26.5 ± 8.5	20.8 ± 10	0.06	−5.7 ± 8.7	30.1 ± 8.5	26.3 ± 6.9	0.08	−3.9 ± 6.2	27.4 ± 8.2	26.1 ± 10	0.57	−1.3 ± 9	0.51 ^#^
Arginine (µU/dL)	39.5 (0, 69.8)	50.1 (0, 100.5)	0.45	16.5 (−69.7, 80.2)	72 (−2.9, 198.9)	62.4 (−1.9, 201.3)	0.33	−3.7 (−14.9, 59.1)	55.4 (0.7, 103.5)	64.2 (0, 76.7)	0.76	7.1 (−71.8, 55)	0.44 ^†^
Macronutrients													
Calories (kcal)	2095 ± 552.1	1779 ± 494	0.07	316 ± 529	2014 ± 248	2152 ± 688	0.5	139 ± 624	2322 ± 837	2294 ± 785	0.95	−28.2 ± 423	0.14 ^#^
Carbohydrates (g)	266 ± 89.5	207 ± 59.7	0.02	59.5 ± 72.4	266 ± 35	265 ± 62.1	0.95	−0.9 ± 52.9	292.8 ± 127	276 ± 118	0.68	−16.6 ±73.6	0.13 ^#^
Protein (g)	78.5 ± 23.9	70.5 ± 24.3	0.32	8 ± 25.7	77.1 ± 21	77.6 ± 22.5	0.94	0.5 ± 23.4	91.1 ± 26.7	92.9 ± 29.8	0.54	1.8 ± 33.9	0.51 ^#^
Fat (g)	82.1 ± 25.5	77.2 ± 25.9	0.61	−4.9 ± 31.9	73.8 ± 23.5	89.7 ± 44.6	0.26	15.9 ± 53.2	83.4 ± 31.6	89.5 ± 32.5	0.39	6.1 ± 37	0.36 ^#^

BMI: body mass index; VO2peak: peak oxygen consumption; HOMA-IR: Homeostasis Model Assessment of Insulin Resistance; HbA1c: glycated hemoglobin; C: cholesterol; HDL: high-density lipoprotein; LDL: low-density lipoprotein; VLDL: very-low-density lipoprotein; PWV: pulse wave velocity; AIx75: augmentation index adjusted to a heart rate of 75 beats per minute; bpm: beats per minute. Data are presented as mean ± SD or median (range), depending on the normality of the distribution. Statistical significance was defined as *p* < 0.05 (*). Analyses were conducted using Student’s paired *t*-test or the Wilcoxon test for within-group comparisons, as appropriate. Between-group differences were analyzed using one-way ANOVA (**^#^**) or the Kruskal–Wallis test (^†^). Post hoc comparisons were conducted using the Bonferroni correction: ^a^ *p* < 0.05 vs L-cit; ^b^ *p* < 0.01 vs. HIIT + placebo; ^c^ *p* < 0.01 vs L-cit; ^d^ *p* < 0.05 vs. HIIT + placebo. Figure 2 shows the significant between-group changes from baseline to 12 weeks for VO2peak, VLDL-C, non-HDL-C, and triglycerides.

**Table 3:** A chi-square test was performed to assess the change in the degree of steatosis across the three intervention groups after 12 weeks.

HIIT + L-cit		Final Steatosis Grade			Total Baseline	*X* ^2^	*p*
		Mild	Moderate	Severe			
Basal Steatosis Grade	Mild	0	1	0	1	3.841	0.43
	Moderate	2	3	2	7		
	Severe	1	0	2	3		
Total Final		3	4	4	11		
HIIT + Pla							
Basal Steatosis Grade	Mild	2	0	1	3	5.667	0.23
	Moderate	2	4	0	6		
	Severe	0	1	0	1		
Total Final		4	5	1	10		
L-cit							
Basal Steatosis Grade	Mild	3	0	0	3	6.514	0.038
	Moderate	4	2	0	6		
	Severe	0	3	0	3		
Total Final		7	5	0	12		

## Data Availability

The dataset used and/or analyzed for the present study is available from the corresponding author on reasonable request. All data underlying the findings of the study are included in this published article.
